# Macrophage-specific autophagy-related gene HSPB8 is involved in the macrophage polarization in atherosclerosis

**DOI:** 10.1186/s12872-023-03158-2

**Published:** 2023-03-18

**Authors:** Juping Wang, Congna Zhao, Baonan Zhang, Xiaoyan Liu

**Affiliations:** 1 Department of Cardiology, Tianjin Beichen Traditional Chinese Medicine Hospital, No.436 Jingjin Road, Beichen District, Tianjin, 300400 P. R. China; 2 Department of Nephrology, Tianjin Beichen Traditional Chinese Medicine Hospital, Beichen District, Tianjin, 300400 P. R. China; 3 Department of Respiratory medicine, Tianjin Beichen Traditional Chinese Medicine Hospital, Beichen District, Tianjin, 300400 P. R. China

**Keywords:** Atherosclerosis, HSPB8, Macrophage polarization, Inflammatory factor

## Abstract

**Background:**

Atherosclerosis (AS) is a chronic inflammatory disease, as a main cause leading to vascular diseases worldwide. Although increasing studies have focused on macrophages in AS, the exact relating mechanism is still largely unclear. Our study aimed to explore the pathogenic role and diagnostic role of macrophage autophagy related genes (MARGs) in AS.

**Methods:**

All datasets were downloaded from Gene Expression Omnibus database and Human Autophagy Database. The differential expression analysis and cross analysis were performed to identify candidate MARGs. GO and KEGG enrichment analyses were conducted to obtain the functional information. Moreover, we analyzed the correlation between target gene and macrophage polarization in AS. The correlation between target gene and plaque instability, different stages of AS were also analyzed.

**Results:**

Compared with normal samples, a total of 575 differentially expressed genes (DEGs) were identified in AS samples. A total of 12 overlapped genes were obtained after cross-analysis of the above 575 DEGs and autophagy related genes (ARGs). Then, 10 MARGs were identified in AS samples, which were significantly enriched in 22 KEGG pathways and 61 GO terms. The expression of HSPB8 was significantly down-regulated in atherosclerotic samples compared with normal samples (with largest fold change). Meanwhile, the proportion of M-CSF in low HSPB8 expression AS group was higher than high expression AS group. Furthermore, the expression of HSPB8 was negatively correlated with most inflammatory factors.

**Conclusion:**

The downregulation of MARG HSPB8 probably involves in the M2 macrophage polarization in AS samples. HSPB8 is a promising diagnostic marker for AS patients.

**Supplementary Information:**

The online version contains supplementary material available at 10.1186/s12872-023-03158-2.

## Background

Atherosclerosis (AS) is a chronic inflammatory disease, as the main cause of vascular diseases around the world [1]. The development of AS has been increasingly indicated to be caused by a combination of genetic and environmental factors, including chronic inflammation, hypertension, diabetes, hypercholesterolemia, sedentary lifestyle and smoking [2–4]. AS is characterized by the formation of atherosclerotic plaques, consisting of necrotic cores, lipids, calcified areas, endothelial cells, inflammatory smooth muscle cells, foam cells and immune cells [5–7]. Once AS plaques are eroded or ruptured, some thrombotic events will be triggered, which may be fatal in severe cases [3]. Macrophages are the vast majority of inflammatory cells in atherosclerotic plaques, determining lesion size, composition, and stability [5, 8]. In the plaques, macrophages contribute to the formation of AS through the uptake of oxidized low-density lipoprotein particles (ox-LDL) and subsequent formation of foam cells [9]. Although increasing studies have focused on macrophages in AS, the exact relating mechanism is still largely unclear.

The role of macrophages in AS is considered to be inseparable from its polarization and phenotypic expression [10]. In the microenvironment of atherosclerotic plaque, the chemokines, lipids, cytokines and other molecules are able to regulate macrophage phenotype and promote the transformation of macrophage to pro-atherosclerotic or anti-atherosclerotic state [5]. Therefore, a better understanding of the phenotypic diversity and function of macrophages is helpful to reveal the detailed macrophage related mechanisms in AS. In atherosclerotic plaques, macrophages originate from the proliferating vascular resident macrophages and the infiltrating monocytes [11]. Monocytes differentiate into macrophages through a variety of pro-differentiation factors, including granulocyte-macrophage colony-stimulating factor (GM-CSF) and macrophage colony-stimulating factor (M-CSF) [12–15]. GM-CSF polarizes macrophages into an inflammatory M1-like phenotype, while M-CSF polarizes macrophages into an anti-inflammatory M2-like phenotype [16]. M1 and M2 macrophages are mutually transformed to control the progress of plaque stability [17].

Moreover, macrophage autophagy also plays an important role in AS [18]. Autophagy is a process of self-protection, which is essential for maintaining cellular homeostasis [19]. Autophagy has been associated with a variety of diseases, including malignant tumors, cardiovascular diseases, immune system disorders and neurodegenerative diseases [20]. Macrophage autophagy defects have been indicated to accelerate AS development by enhancing foam cell formation, cell death, and inflammation [21]. Although the exact mechanism of macrophage in the regulation of AS is unclear, certain autophagy related genes seem to be key factors contributing to AS. For example, it has been reported that in AS mouse models, macrophages lacking of autophagy-related gene 5 (ATG5) could promote the oxidative stress and plaque necrosis [22]. Moreover, there are also some other markers for AS. Endothelin-1 and C reactive protein have recently been suggested to serve as promising markers for restenosis in AS obliteran patients [23]. Whereas, the current biomarkers for AS are far from meeting the clinical requirements. HSPB8 encodes the small heat shock protein B8 (HSPB8), associating with various cellular functions, including autophagy, cytoskeleton stabilization, apoptosis, oxidative stress, proliferation and differentiation [24]. Meanwhile, aberrant HSPB8 expression has involved in causing a variety of diseases, such as breast cancer, lung cancer, glioblastoma and hepatocarcinoma [24–27]. However, the pathogenic role or diagnostic role of HSPB8 in AS has not been clarified yet as far as we know.

In this study, based on public data in multiple databases, we screened macrophage-specific autophagy-related genes in AS samples, meanwhile the hub gene was then identified and subjected to further analysis. Our findings may provide a new target for the diagnosis and treatment of AS.

## Materials and methods

### Data source

The GSE100927 dataset contained 104 AS samples (Agilent-039494 SurePrint G3 Human GE v2 8 × 60 K Microarray) and GSE43292 dataset included 64 AS samples (Affymetrix Human Gene 1.0 ST Array), which were downloaded from Gene Expression Omnibus (GEO; https://www.ncbi.nlm.nih.gov/geo/) database. Autophagy related genes (ARGs) were obtained from Human Autophagy database (http://www.autophagy.lu/index.html) (Table S1). Additionally, GSE120521 (Illumina HiSeq 2500), GSE18275 (RNG-MRC_HU25k_EVRY), GSE28829 (Affymetrix Human Genome U133 Plus 2.0 Array) and GSE23314 (Rosetta / Merck Human 44k 1.1 microarray) datasets were also downloaded from GEO database.

### Differentially expressed gene analysis

The “limma” R package (version 4.2.0, the same below) was used to screen differentially expressed genes (DEGs) from GSE100927 dataset with |Log2FC| > 1 and P value ≤ 0.05 [28].

### Functional enrichment analyses

Based on the mutual DEGs, the “clusterProfiler” R package [29] was utilized to conduct the GO (Biological Process, Molecular Function and Cellular Component) and KEGG (Kyoto Encyclopedia of Genes and Genomes) pathway enrichment analysis. The P value < 0.05 was considered statistically significant.

### The correlation between target gene and macrophage polarization in AS

To further evaluate the differences of GM-CSF and M-CSF in macrophages between high and low target gene expression AS samples, we divided the samples in the GSE18275 into different groups according to the median expression of target gene. As AS is accompanied by local production and release of inflammatory mediators [30], we analyzed the correlation between the target gene expression and interferon/receptor and interleukin/receptor mRNA expression (such as IFNAR2, IL10RA, IL10RB, IL32, IL4R, IL-4, IL-10, IL-6, IL-1B and TNF).

### The correlation between target gene and plaque instability, different stages of AS

The correlation between target gene and plaque instability was studied in dataset GSE120521. In addition, dataset GSE28829 was used to explore the expression of target gene at different stages of AS. Receiver operating characteristic (ROC) analysis was employed to determine the diagnostic value of target gene.

## Results

### Identification of candidate macrophage autophagy related genes in AS

**Fig. 1:**
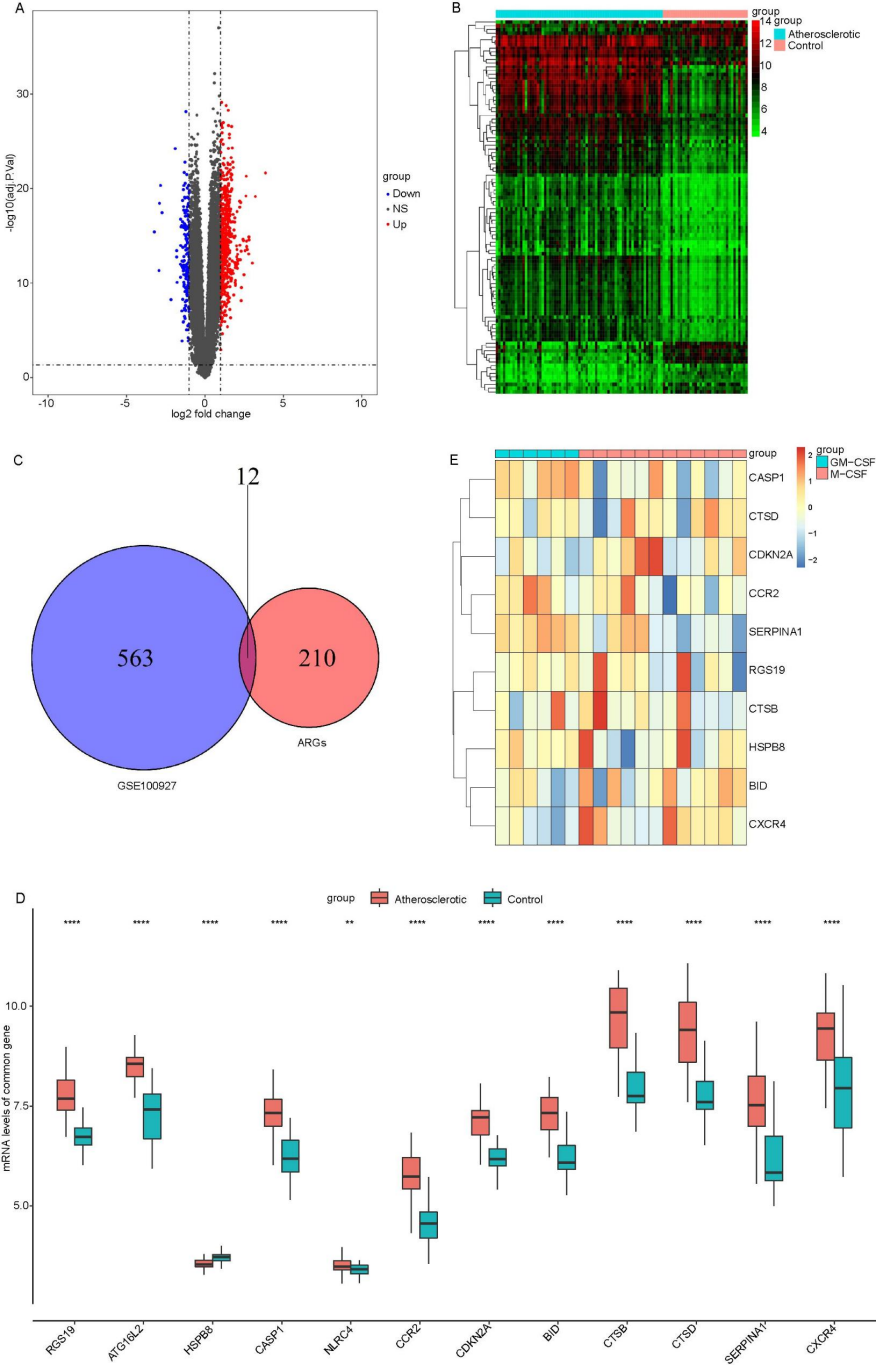
**The results of differential expression analyses.** A-B. Volcanic map and heat map of differentially expressed genes, respectively. C. Venn diagram showing overlapped genes. D. The expression of 12 overlapped genes in AS. E. Totally 10 macrophage autophagy related genes (MARGs) were identified in AS samples

Compared with normal samples, totally 575 DEGs were identified in AS samples basing on dataset GSE100927, including 422 up-regulated genes and 153 down-regulated genes (Fig. 1A and B, Table S2). A total of 12 overlapped genes were obtained after cross-analysis of the above 575 DEGs and ARGs (Fig. 1C), and the expression of the 12 overlapped genes in AS were shown in Fig. 1D. Among which, 10 genes were expressed in macrophages, based on the macrophage related mRNA profile in GSE18275 (Fig. 1E). Thus, 10 macrophage autophagy related genes (MARGs) were identified in AS samples, including CASP1, CTSD, CDKN2A, CCR2, SERPINA1, RGS19, CTSB, HSPB8, BID and CXCR4.

### Results of GO and KEGG functional enrichment analysis

**Fig. 2:**
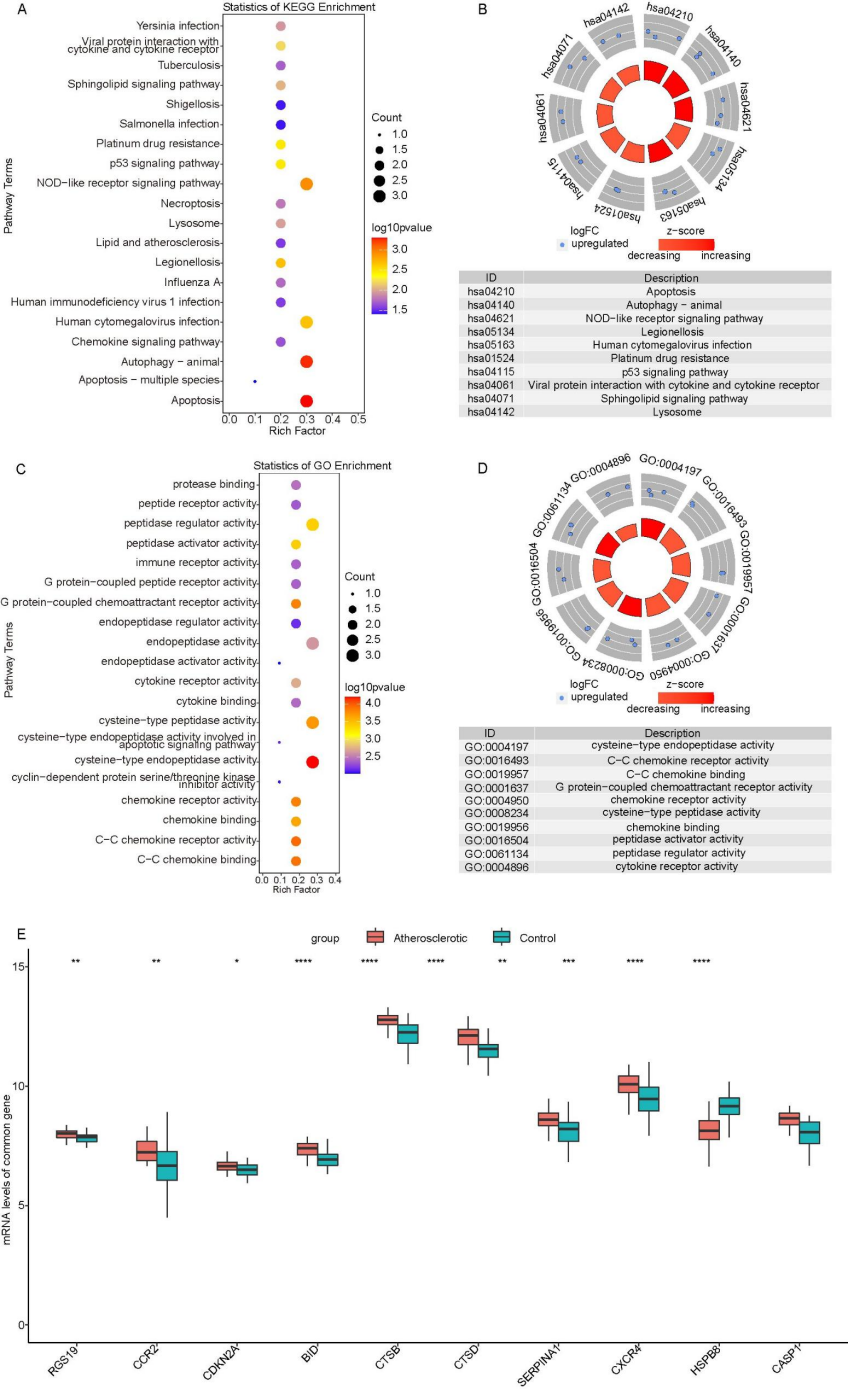
**The results of GO and KEGG functional enrichment analysis.** (A) Bubble map of significant KEGG pathways. (B) Loop map of significant KEGG pathways. (C) Bubble map of significant GO terms. (D) Loop map of significant GO terms. (E) The expression of 10 MARGs in an independent cohort GSE43292.

We conducted GO and KEGG enrichment analyses on the above 10 MARGs. These 10 MARGs were significantly enriched in 22 KEGG pathways (Table S3)(The pathways were obtained based on KEGG [31–33]) and 61 GO terms (Table S4). Significant KEGG pathways included apoptosis, autophagy, chemokine and lipid and atherosclerosis signaling pathways, and the top 20 pathways were shown in Fig. 2A and B. Significantly enriched GO terms included chemokine receptor activity and tumor necrosis factor receptor pathways, and the most significantly enriched 20 GO terms were displayed in Fig. 2C and D.

Moreover, we performed a differential expression analysis on these 10 MARGs in an independent cohort GSE43292. The results showed that the fold change of HSPB8 was the largest (Fig. 2E, Table S5, log FC = -0.938204687, P < 0.0001). Therefore, HSPB8 was selected for our subsequent analysis in AS.

### HSPB8 was correlated with the macrophage polarization in AS

**Fig. 3:**
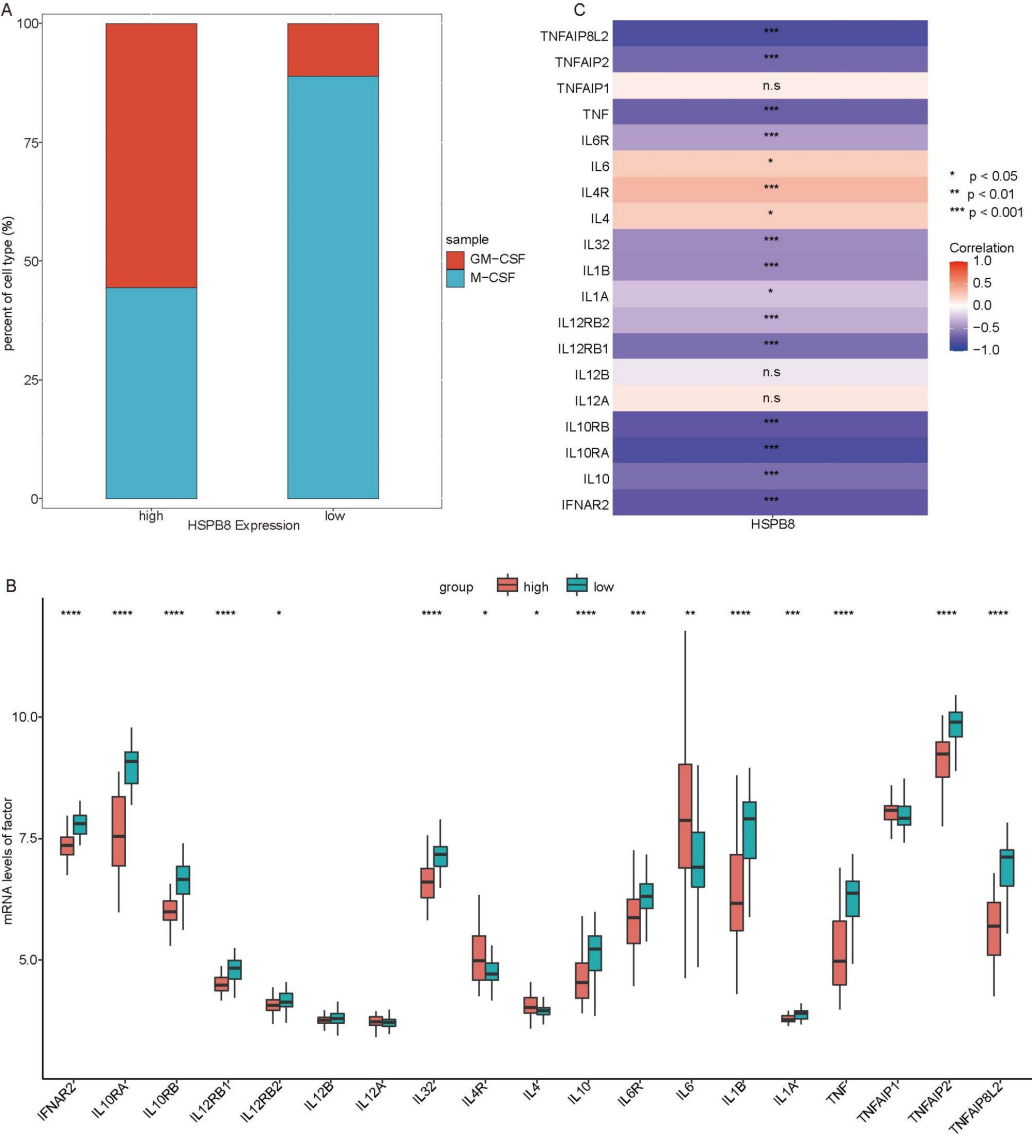
**Polarization analysis of macrophages.** (A) The proportion of macrophage differentiation factors, M-CSF and GM-CSF. (B) Bar chart of interferon/receptor and interleukin/receptor. (C) Heat map of the correlation between HSPB8 and interferon/receptor and interleukin/receptor

Given the significant effects of different subtypes of macrophages on AS patients, the proportions of M-CSF and GM-CSF in AS samples with differential MARG HSPB8 expression were analyzed. All AS samples in dataset GSE18275 were divided into high and low HSPB8 expression AS groups, according to the median expression level of HSPB8. We found that the proportions of M-CSF in low HSPB8 expression group were significantly higher than that in high HSPB8 expression group (Fig. 3A). In low HSPB8 expression AS group, significantly higher M-CSF proportions indicated that anti-inflammatory M2 macrophage was the predominant subtype (Fig. 3A).

The correlation between HSPB8 and various inflammatory factors was then evaluated based on GSE100927. We found that IL4, IL4R and IL6 in the HSPB8 high expression group was significantly up-regulated compared with the HSPB8 low expression group, while IFNAR2, IL10RA, IL10RB, IL12RB1, IL12RB2, IL32, IL10, IL6R, IL1B, IL1A, TNF, TNFAIP2 and TNFAIP8L2 were significantly down-regulated (Fig. 3B). Subsequent correlation analysis showed that HSPB8 was negatively correlated with IFNAR2, IL10, IL10RA, IL10RB, IL12RB1, IL12RB2, IL1A, IL1B, IL32, IL6R, TNF, TNFAIP2, TNFAIP8L2 (PCC < -0.4, P < 0.05), but HSPB8 was positively correlated with IL4, IL6 and IL4R (PCC > 0.4, P < 0.05) (Fig. 3C).

### The diagnostic value of HSPB8 in AS patients

**Fig. 4:**
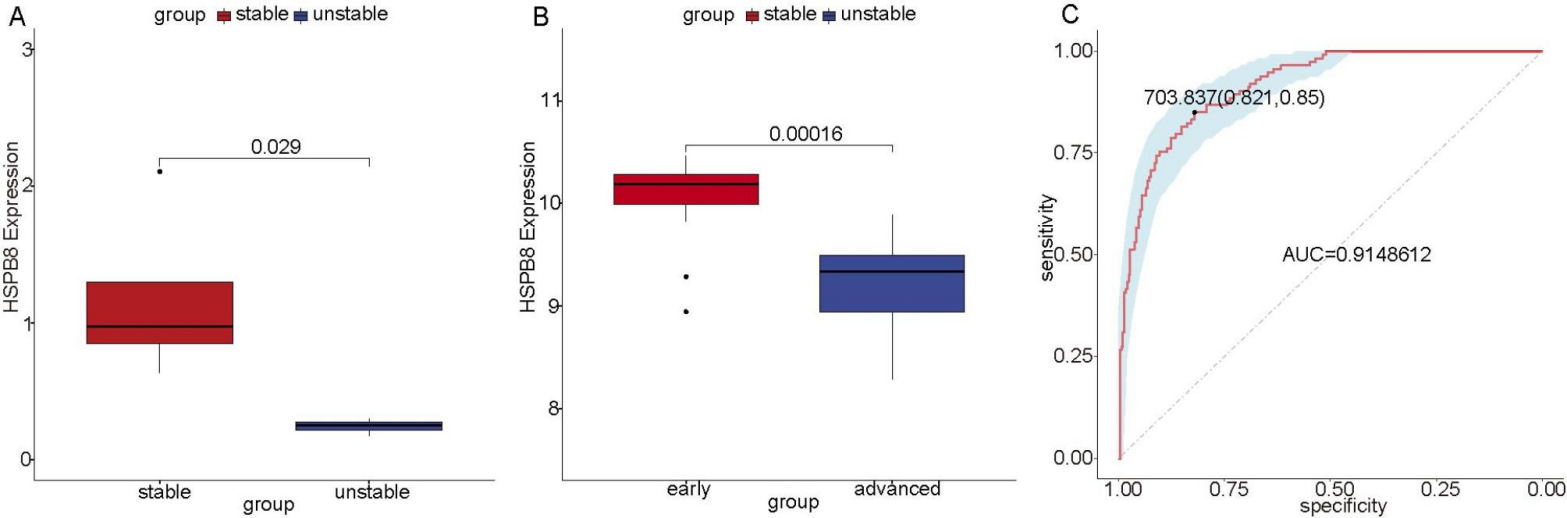
**The expression of HSPB8 in different AS samples.** (A) HSPB8 expression in AS samples with sable and unstable plaques. (B) HSPB8 expression in AS samples from different stages. (C) ROC curve verification of HSPB8.

Subsequently, we have also evaluated the diagnostic value of HSPB8 in AS patients. There are two types of atherosclerotic plaques, stable plaques and unstable plaques. The fibrotic cap of the stable plaques is thick and the lipid core is small [34]. In contrast, unstable plaques are prone to rupture, characterized by more macrophages, thin fibrotic cap, large necrotic center, plaque erosion, calcified nodules [35, 36]. Therefore, the risk coefficient of unstable plaques is higher, which is more likely to cause myocardial infarction and cerebral infarction. After analyzing the AS data including clinical plaque information (GSE120521), we found that significantly lower HSPB8 expression was observed in AS with unstable plaques, compared with AS with stable plaques (Fig. 4A), indicating a higher risk of AS subsequent disease. Moreover, compared with early AS, there was significantly lower HSPB8 expression in advanced AS (GSE28829, Fig. 4B), which also implied a higher possibility of subsequent diseases. Furthermore, the results of ROC analysis suggested that HSPB8 could be used as a diagnostic marker of AS (area under curve (AUC) = 0.9148612, Fig. 4C).

## Discussion

AS is a complex disease process and the number one killer worldwide [2]. In our study, we explored the potential pathogenic mechanism of AS basing on multiple AS public datasets. The expression of HSPB8 was significantly down-regulated in AS samples compared with normal samples. Meanwhile, the proportion of M-CSF in low HSPB8 expression group was higher than that in high expression group. Moreover, the expression of HSPB8 was negatively correlated with most inflammatory factors. In brief, our study suggested that low HSPB8 expression probably promoted the M2 macrophage polarization in AS samples.

After the analysis of data in GEO and Human Autophagy Database, 10 candidate MARGs were identified in AS, then HSPB8 with the largest fold change exhibiting lower expression in AS was selected for our subsequent analysis. In some malignancies, HSPB8 has been demonstrated to be silenced by DNA methylation [37, 38], while the underlying epigenetic modification of HSPB8 in AS remains largely unknown, deserving further exploration. In addition, it has been indicated that HSPB8 activation plays a crucial role in controlling inflammation and promoting tissue repair [39]. HSPB8 is also named small stress protein like-protein (HSP22) [40]. The overexpression of HSP22 has been reported to protect endothelial damage by inhibiting inflammation and oxidative stress [41]. Whether low expression of HSPB8 exerts an opposite role in endothelial damage of AS can not be concluded from present work, but it deserves further investigation. Yu et al. have suggested that HSP22 significantly inhibited endothelial cell activation and vascular lesions [42]. It is well known that severe reduction of coronary blood flow causes ischemic injury in the heart [43]. Recent studies have revealed the potential of HSP22 in reducing ischemic injury [44, 45]. The crucial role of HSPB8 in vascular diseases partly supported our findings in AS, and more related details should be clarified in future.

In AS, the proportion of M-CSF was higher in the low HSPB8 expression group, that is, a large number of macrophages were polarized into M2 macrophages, which were consistent with previous findings [46, 47]. It is well known that macrophage polarization plays a pivotal role throughout the progression of AS [47]. M1 and M2 macrophages play different roles in inflammation, depending on microenvironmental stimuli [48, 49]. When changes in microenvironment are sensed, macrophages can switch between M1 and M2 phenotypes [50]. Furthermore, both M1 and M2 macrophages accumulate along with the degree of lesion in atherosclerotic plaque [50]. Early atherosclerotic plaques were mainly infiltrated by M2 macrophages, but with the progress of plaques, M1 macrophages gradually increased and occupied the major position [51, 52]. Gong et al. have documented that in the murine model, M1 macrophages were enriched in the unstable plaques, while M2 macrophages were reduced [46]. However, little is known about the dynamic phenotypic changes of macrophages at different stages of AS, and there is the possibility of replacement or local proliferation in macrophages [50, 53, 54]. Thus, more details concerning the mechanism of macrophage polarization in AS need to be further explored in the near future.

Additionally, AS is usually accompanied by chronic, low-grade inflammatory reaction [55]. We analyzed the differences in inflammatory factors between groups with different levels of HSPB8 expression. In HSPB8 high expression group, IL4, IL4R and IL6 were significantly upregulated, but IFNAR2, IL10, IL10RA, IL10RB, IL12RB1, IL12RB2, IL1A, IL1β, IL32, IL6R, TNF, TNFAIP2 and TNFAIP8L2 were significantly downregulated. Recent evidence has indicated that the important effect IL-1β exerted on AS [56, 57]. IL-1β induced inflammatory reaction in endothelial cells and promoted the accumulation of inflammatory cells in blood vessels, which usually occurred at the beginning of AS [58]. As a pro-inflammatory factor, IL-32 is involved in the inflammatory cascade that leads to AS and further promotes plaque instability [59]. Jin et al. have documented TNFAIP2 promotes atherogenesis by enhancing oxidative stress induced inflammation [60]. All the above studies indicated that promoting some inflammatory factors may accelerate the development of AS. In general, M2 macrophage polarization inhibits the expression of inflammatory factors to suppress the disease [46]. But a study showed that IFNc/LPS stimulates M-CSF to polarize macrophages toward M1-like phenotype [61]. Therefore, this may explain the upregulation of inflammatory factors in the present study. On the other hand, our results have implied the strong correlation between low HSPB8 expression and high proportion of M-CSF as well as inflammatory factors. Although in this work, whether AS patients with differential HSPB8 expression showed different responses in the treatment can not be concluded temporarily, our findings proposed an interesting probability involving the personalized treatment of AS patients.

Although the potential role of HSPB8 involving macrophage polarization and autophagy in AS has been explored in this work for the first time, there were still some limitations in our study. Firstly, though our work has included as much data as possible, our present results were limited by publicly obtained data. More AS macrophage polarization related expression profiles would be more conducive to a deepening analysis. Moreover, the diagnostic role of HSPB8 in AS should be further validated in a larger sample size.

## Conclusion

In summary, based on large amounts of public data, MARG HSPB8 has been identified as a diagnostic marker for AS patients for the first time. Meanwhile, HSPB8 expression is associated with the macrophage polarization and inflammatory factors in AS, which probably accelerates the progression of AS indirectly. Although more details still deserve to be further investigated, our findings provide novel reference information regarding the macrophage polarization related mechanisms and treatment strategies for AS.

## Electronic supplementary material

Below is the link to the electronic supplementary material.

Additional File: Table S1: The autophagy related genes downloaded from Human Autophagy database

Additional File: Table S2: The detailed list of DEGs identified between AS and normal samples

Additional File: Table S3: All significantly enriched KEGG pathways

Additional File: Table S4: All significantly enriched GO terms

Additional File: Table S5: The expression difference of 10 MARGs between AS and normal samples

## Data Availability

The datasets analyzed during the current study are available in Gene Expression Omnibus (GEO; https://www.ncbi.nlm.nih.gov/geo/) database[GSE100927, GSE43292, GSE120521, GSE18275, GSE28829, GSE23314] and Human Autophagy database (http://www.autophagy.lu/index.html).
